# Timing of renal replacement therapy initiation by AKIN classification system

**DOI:** 10.1186/cc12593

**Published:** 2013-04-02

**Authors:** Tacyano T Leite, Etienne Macedo, Samuel M Pereira, Sandro RC Bandeira, Pedro HS Pontes, André S Garcia, Fernanda R Militão, Irineu MM Sobrinho, Livia M Assunção, Alexandre B Libório

**Affiliations:** 1 Division of Nephrology, General Hospital of Fortaleza, Rua Ávila Goulart no. 900, 60175-295, Fortaleza, Brazil; 2 Nephrology Department, University of São Paulo, Av. Dr. Eneas Carvalho de Aguiar no. 255, 05403-000, São Paulo, Brazil; 3 Medical Course, UNIFOR, Av. Washington Soares no. 1321, 60811-905, Fortaleza, Brazil; 4 Internal Medicine, Faculdade de Medicina, Universidade Federal do Ceará, Rua Alexandre Baraúna no. 900, 60430-160, Fortaleza, Brazil; 5 Nephrology, Cancer Institute of Ceará, R. Papi Júnior no.1222, 60430-230, Fortaleza, Brazil

## Abstract

**Introduction:**

Previous studies using Acute Kidney Injury Network (AKIN)/RIFLE criteria to classify early initiation of renal replacement therapy (RRT) have defined it as the therapy started in less severe AKIN/RIFLE stages. Generally, these studies failed in demonstrating measurable benefits.

**Methods:**

We compared RRT initiation in critically ill patients and defined early or late RRT in reference to timing after stage 3 AKIN was met: patients beginning RRT within 24 hours after acute kidney injury (AKI) stage 3 were considered early starters. AKIN criteria were evaluated by both urine output (UO) and serum creatinine (sCr) and patients with acute-on-chronic kidney disease were excluded. A propensity score methodology was used to control variables.

**Results:**

A total of 358 critically ill patients were submitted to RRT. Only 150 patients with pure AKI at stage 3 were analyzed. Mortality was lower in the early RRT group (51.5 vs. 77.9%, *P *= 0.001). After achieving balance between the groups using a propensity score, there was a significant 30.5 (95% confidence interval [CI] 14.4 to 45.2%, *P *= 0.002) relative decrease of mortality in the early RRT group. Moreover, patients on the early RRT group had lower duration of mechanical ventilation, time on RRT and a trend to lower intensive care unit (ICU) length of stay.

**Conclusions:**

For the first time, AKIN was used with UO criterion to evaluate early and late RRT. Using a time-based approach could be a better parameter to access the association between RRT initiation and outcomes in patients with AKI.

## Introduction

Acute kidney injury is a common occurrence in critically ill patients, with incidence rates of occurrence varying from 5 to 60% [1,2] and a trend towards higher rates (30 to 60%) when using the risk, injury, failure, loss of kidney function, end stage renal failure (RIFLE) or Acute Kidney Injury (AKI) Network (AKIN) classification. Acute kidney injury is an independent risk factor for increased morbidity and mortality [3,4]. Renal replacement therapy (RRT) is necessary in about 6% of critically ill patients, according to a large multinational, multicenter survey [1]. Generally, RRT is provided as supportive treatment to AKI patients, preventing additional disorders (hypervolemia, metabolic acidosis, progressive uremia, and hyperkalemia).

Data from mainly observational studies have suggested that early RRT in critically ill patients with AKI may have a beneficial impact on survival [5,6]. However, in addition to the lack of large randomized clinical trials assessing early dialysis indication, there is a broad variation in the criteria used to classify early or late RRT. In a review of 16 studies comparing early vs late dialysis, metabolic markers (serum urea or hyperkalemia) were the most frequently used parameters (*n *= 8) [7-13]. Reduced urine output (*n *= 3) [14-16] or timing from ICU admission to RRT start (*n *= 2) [17,18] have also been used to define early dialysis. RIFLE criteria have been used in three other studies to define early dialysis [19-21].

Although the RIFLE criteria were developed and validated for AKI diagnosis and evaluation of severity, and not a parameter to guide RRT initiation, some observational studies [19-21] have investigated whether initiating RRT in less severe renal injury (that is, RIFLE classification -0 or R) can be associated with a better survival rate. Although one study that analyzed AKI after major abdominal surgery demonstrated benefits [20], two others studies evaluating mainly patients with sepsis demonstrated similar outcomes with either early or late RRT [19,21]. However, two problems can make the analysis of survival benefits in early dialysis difficult when using this definition: (1) patients at less severe AKI stages initiating RRT may have severe metabolic disorders (that is, metabolic acidosis, or hyperkalemia) in spite of limited renal injury, or even no AKI (RIFLE classification -0); (2) when using RIFLE/AKIN criteria, patients with chronic kidney disease (CKD) must have significant metabolic disorders to require RRT before classification with the maximum severity grade, as opposed to those with previous normal previous renal function. Moreover, no study has evaluated urinary output (UO), making the degree of AKI severity largely dependent on baseline serum creatinine (sCr), and there is no information on the timing of RRT initiation after diagnosis of severe AKI.

In the present study, a different approach using AKI severity classification was used. Our objective was to determine whether the timing of RRT earlier or later than 24 hours after classification as AKIN stage 3, is associated with hospital mortality in critically ill patients. Moreover, we excluded acute-on-chronic kidney injury (AKI-CKD) patients.

## Materials and methods

This is a retrospective analysis of a prospective cohort of consecutive critically ill adult patients (> 18 years of age) undergoing renal replacement therapy. The study was carried out at referral centers (General Hospital of Fortaleza and Haroldo Juaçaba Hospital) in Fortaleza, Brazil. These hospitals comprise four ICUs with a total of 43 beds. All patients needing RRT from January 2010 to December 2011 were initially included. We excluded patients undergoing maintenance RRT, with renal transplantation, with RRT initiated before ICU admission, those who needed RRT before being classified as AKIN stage 3 and with an ICU stay of less than 24 h. Patients who did not have sCr less than or equal to 1.4 mg/dL during their hospital stay were considered as having previous CKD and were excluded. This definition was used instead of glomerular filtration rate (GFR) because it is the highest level where AKI stage 3 could be diagnosed by a three-fold increase in reference sCr and not by an acute increment of 0.5 mg/dL in patients with sCr more than or equal to 4.0 mg/dL, as discussed below. All data, including UO at each 6-hour interval, were prospectively collected during the ICU stay by a group of attendant physicians and nurses after a standardized database record was implemented. Data preceding ICU admission were obtained from medical records. We did not have access to UO data before ICU admission. The Institutional Ethical Committee of the Hospital Geral de Fortaleza approved the analysis of data and agreed that informed consent was unnecessary due to the purely observational and non-interventional nature of this study (Resolução CNS 196/96).

### Clinical and laboratory parameters

Data variables included demographic characteristics, comorbidities, surgical or medical ICU admission and the indications for RRT. Biochemistry data such as complete blood cell count, serum urea (sUr), sCr, blood gas analyses, serum total bilirubin and serum potassium were recorded upon ICU admission and RRT initiation. The clinical parameters and severity score were also recorded. The clinical parameters included heart rate, systolic and diastolic blood pressure, partial pressure of arterial blood gas oxygen and fraction of inspired oxygen. Urine output was measured each 2-hour interval during the ICU stay. Severity scores included the Glasgow Coma Scale (GCS), Acute Physiology and Chronic Health Evaluation II (APACHE II) score and Simplified Acute Physiology Score III (SAPS III). The use of mechanical ventilation and need for vasopressor drugs were recorded. Definitions were made as follows: hypertension was defined as blood pressure above 140/90 mmHg or use of anti-hypertension agents; diabetes was defined as previous use of insulin or oral hypoglycemic agents; sepsis was defined as the presence of both infection and systemic inflammatory response syndrome [22]. All sCr levels measured from hospital admission until the start of RRT were recorded. At hospital discharge, the GFR of survivors was estimated by the CKD-Epidemiology Collaboration (EPI) equation.

The indications for RRT were: (1) azotemia (sUr > 150 mg/dL) with uremic symptoms; (2) oligoanuria (urine output < 100 ml every twelve hours) refractory to diuretics; (3) hyperkalemia (sK^+ ^> 5.5 mmol/L) refractory to medical treatment, and (4) metabolic acidosis (pH < 7.2 and serum bicarbonate < 16 m Eq/L in arterial blood gas).

### Acute kidney injury classification

Diagnosis and severity of AKI was defined by the AKIN classification system, using the worst criteria (sCr increment or reduced UO). The reference sCr was the lowest achieved during hospital stay before RRT start. Time from AKI stage 3 diagnosis to RRT start was recorded. Patients with more than one AKI episode during their hospital stay had only the first RRT-related AKI episode recorded. Patients initiating RRT less than 24 h after reaching AKIN stage 3 were included in the early RRT group and those after 24 h were included in the late RRT group.

### Renal replacement therapy modality

Prescription and initiation of RRT was performed by two groups of nephrologists who did not work with the present researchers. When patients were receiving vasopressor drugs, sustained low efficiency RRT (SLED) was performed; otherwise, conventional intermittent hemodialysis was performed. For SLED, the blood flow and dialysate flow rates were 200 ml/minute and 300 ml/minute, respectively, and the duration was 8 h. Conventional intermittent hemodialysis was performed for 4 h with a dialysate flow of 500 ml/minute, and blood flow of 300 ml/minute. As hemodynamics changed during the ICU stay, patients changed between RRT modalities.

### Outcomes

The endpoint of this study was in-hospital mortality. The survival period was calculated from RRT initiation to mortality in non-survivors or to hospital discharge in survivors. Secondary endpoints included ICU length of stay (LOS), days on mechanical ventilation and time on RRT.

### Statistical analysis

Descriptive statistics are expressed as mean ± SD, or as absolute frequency and percentage, according to continuous or categorical data, respectively. All variables were tested for normal distribution using the Kolmogorov-Smirnov test. The unpaired Student's *t*-test was applied to compare continuous variables and normally distributed data when appropriate. Categorical data were tested using the chi-square test.

Kaplan-Meier curves obtained with the log-rank test were plotted to demonstrate the differences in patient survival between the two groups (early RRT vs late RRT). A propensity score methodology was used to control for bias due to selection of patients placed on early RRT, using the inverse probability of treatment weighting (IPTW) method. This method has been shown to have superior performance in estimation of risk differences in observational studies [23]. First, a propensity score model included demographic, clinical and laboratory data at ICU admission and at the day of RRT start that could have influenced the decision of placement on early RRT. This estimated the probability for each study subject to be placed on early RRT. Then, the contribution of each subject was weighted by 1/propensity score for the early RRT group, and by 1/(1-propensity score) for the delayed late RRT group. These weights assured that for each combination of the covariates used in the propensity score model, the sum of the contributions of all subjects was equal. All pre-RRT variables were balanced between groups. Covariate balance after the IPTW weighting was assessed by computing their standardized differences, and groups were considered balanced if the standardized differences of all covariates were < 0.25 [24] and no further adjustment was required for the outcome analysis. Short-term outcome variables were compared using IPTW-weighted regression models. Results were expressed as absolute and relative average treatment effect. Statistical significance was set at *P *< 0.05. Analyses were performed with SPSS 19.0 for Windows and R software version 2.10.1 for Windows, using the Design and Survey packages [25].

## Results

**Figure 1:**
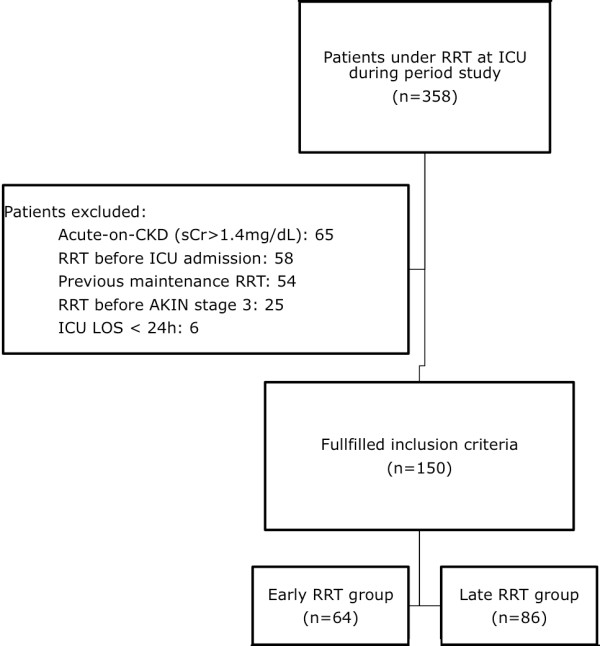
**Patient identification and selection approach**. RRT, renal replacement therapy; CKD, chronic kidney disease; AKIN, Acute Kidney Injury Network; LOS, length of stay.

During the study period, 358 patients were submitted to the ICU for RRT. Overall, 143 were excluded from analysis. Only 25 patients were excluded because RRT was initiated before stage 3 AKI was reached. Of the 215 patients available for analysis, 150 had AKI without previous CKD and 65 had AKI-CKD (Figure 1).

### Patients' characteristics

Overall, the mean age of patients was 55.5 ± 19.1 years and 84 (57.5%) were male. Most patients (86.0%) were septic. Overall, in-hospital mortality was 72.0%. Complete clinical and laboratory data are shown in Table 1. Sixty-four patients (42.6%) were in the early RRT group. Among patients in the late RRT group, 41 (47.6%) initiated RRT 24 to 48 h after AKI stage 3 was reached and 21 (24.4%) after 48 to 72 h.

**Table 1:** Patients' characteristics before and after inverse probability weighting

		Before weighting				After inverse probability of treatment weighting	
		
	AKI (*n *= 150)	ED group (*n *= 64)	LD group (*n *= 86)	Standardized difference	*P*-value	Standardized difference	*P*-value
Age, years	55.5 ± 19.1	54.6 ± 19.7	56.2 ± 18.4	0.08	0.604	0.09	0.586
Sex, male, n (%)	84 (57.5)	35 (54.7)	51 (59.3)	0.09	0.539	0.01	0.960
Diabetes mellitus, n (%)	34 (22.7)	14 (21.9)	20 (23.2)	0.03		0.05	0.812
Hypertension, n (%)	67 (44.7)	27 (42.1)	40 (46.5)	0.09		0.07	0.698
Lower sCr, mg/dL	0.90 ± 0.30	0.96 ± 0.30	0.87 ± 0.29	0.29	0.056	0.03	0.849
Surgical ICU admission, n (%)	27 (18.0)	32 (50)	45 (52.3)	0.04	0.778	0.13	0.409
Sepsis diagnosed, n (%)	129 (86.0)	56 (87.5)	73 (84.9)	0.07	0.648	0.13	0.402
**Data at ICU admission**							
Vasoactive drugs, n (%)	116 (77.3)	49 (76.6)	67 (77.9)	0.03	0.846	0.15	0.338
Systolic blood pressure, mmHg	101.0 ± 22.9	91.8 ± 20.2	108.6 ± 22.5	0.73	0.001	0.17	0.174
Diastolic blood pressure, mmHg	59.4 ± 14.9	55.5 ± 13.4	62.5 ± 15.3	0.13	0.033	0.14	0.201
Heart rate, bpm	101.1 ± 23.5	104.7 ± 21.5	99.3 ± 24.8	0.23	0.160	0.02	0.872
Hemoglobin, g/dL	10.1 ± 2.2	9.8 ± 2.4	10.5 ± 2.1	0.29	0.069	0.14	0.367
White blood cells, x10^3^/mm^3^	17.0 ± 8.6	15.9 ± 6.6	17.5 ± 7.4	0.07	0.643	0.11	0.659
Platelet count, x10^3^/mm^3^	230.4 ± 121.7	235.3 ± 131.2	226.4 ± 120.2	0.06	0.724	0.15	0.349
Creatinine, mg/dL	2.11 ± 1.22	2.0 ± 0.9	1.7 ± 0.8	0.04	0.791	0.11	0.357
Urea, mg/dL	78.7 ± 43.1	82.7 ± 44.1	75.4 ± 41.8	0.12	0.481	0.09	0.560
Potassium, mEq/L	4.4 ± 2.1	4.7 ± 1.4	4.2 ± 0.9	0.14	0.394	0.03	0.494
Bicarbonate, mEq/L	17.7 ± 5.7	16.7 ± 5.5	18.5 ± 5.9	0.30	0.071	0.19	0.250
GCS score	12.8 ± 2.7	12.8 ± 3.1	12.8 ± 2.5	0.01	0.986	0.17	0.331
APACHE II score	18.8 ± 6.1	19.2 ± 6.3	18.7 ± 5.9	0.08	0.520	0.03	0.848
SAPS III score	61.1 ± 11.9	62.1 ± 11.7	60.2 ± 12.0	0.15	0.327	0.20	0.227
**Data at RRT start**							
Creatinine, mg/dL	2.93 ± 1.82	2.7 ± 1.1	2.8 ± 1.5	0.04	0.780	0.03	0.855
Urea, mg/dL	106.7 ± 64.8	100.1 ± 54.9	108.2 ± 55.6	0.17	0.461	0.02	0.908
Potassium, mEq/L	5.3 ± 2.0	4.9 ± 1.9	5.1 ± 2.1	0.11	0.499	0.15	0.392
Bicarbonate, mEq/L	16.2 ± 4.4	14.4 ± 3.9	17.2 ± 4.3	0.64	< 0.0001	0.14	0.501
APACHE II score	20.2 ± 6.3	20.3 ± 6.8	20.1 ± 6.2	0.06	0.637	0.05	0.737
SAPS III score	62.6 ± 12.3	63.2 ± 11.8	62.7 ± 11.6	0.10	0.469	0.14	0.298
**Indications for dialysis**							
Azotemia, n (%)	27 (18.0)	7 (10.9)	20 (23.3)	0.34	0.052	0.06	0.715
Oligoanuria, n (%)	118 (78.7)	53 (82.8)	65 (75.6)	0.18	0.285	0.20	0.305
Metabolic acidosis, n (%)	96 (64.0)	46 (71.9)	50 (58.1)	0.29	0.083	0.17	0.358
Hyperkalemia, n (%)	21 (14.0)	4 (6.2)	17 (19.8)	0.41	0.018	0.13	0.402

Results are presented as mean (± SD) unless or number (%) of patients. AKI, acute kidney injury; CKD, chonic kidney disease; RRT, renal replacement therapy GCS, Glascow Coma Scale; APACHE, Acute Physiology and Chronic Health Evaluation; SAPS, Simplified Acute physiology Score; sCr, serum creatinine; ED, early dialysis; LD, late dialysis.

**Figure 2:**
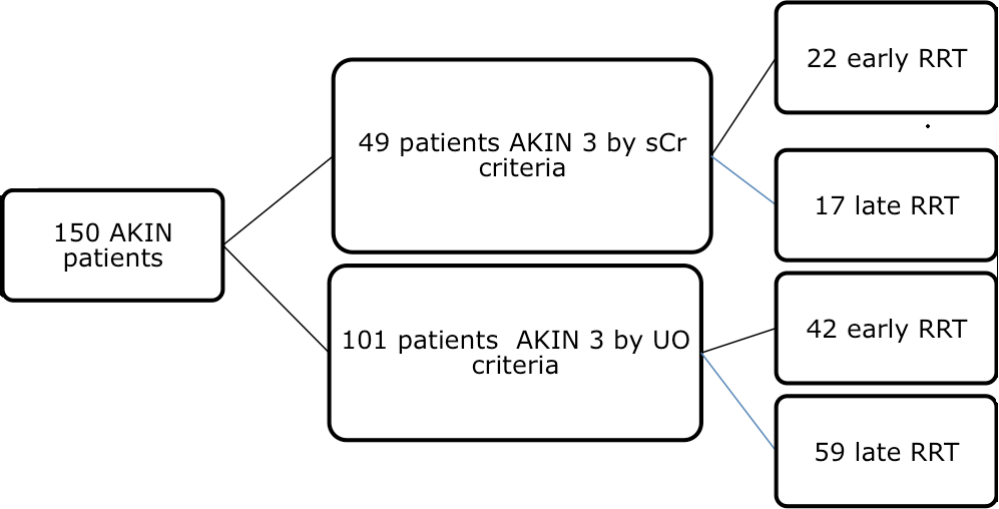
**Flowchart of patients achieving Acute Kidney Injury Network stage 3 by serum creatinine or urinary output**. AKIN, Acute Kidney Injury Network; sCr, serum creatinine; UO, urinary output; RRT, renal replacement therapy.

**Figure 3:**
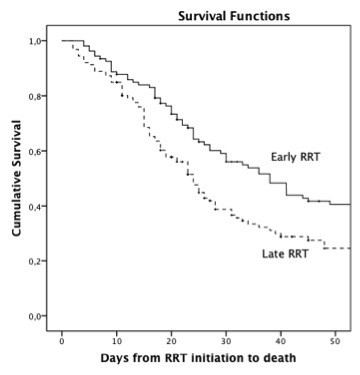
**Cumulative patient survival between early and late renal replacement therapy groups**. Log rank test, *P *= 0.031. RRT, renal replacement therapy.

Patients initiating RRT earlier had lower blood pressure at ICU admission, were more acidotic and had lower hemoglobin levels. There was a trend towards RRT initiation due to acidosis in early RRT group and due to azotemia and hyperkalemia in late RRT group (Table 1). In-hospital mortality was lower in patients starting RRT earlier (51.5 vs 77.9%, *P *= 0.001) (Table 2). Mortality rates according with early or late RRT were very similar if patients reached AKI stage 3 first according to sCr vs UO criteria (Figure 2). Survival probabilities are shown in Figure 3. The log rank test of Kaplan-Meier curves showed a significant difference between groups (log rank *P *= 0.031). At discharge, no AKI patient without CKD was RRT-dependent and 18 patients (60.7%) had a GFR > 60 mL/min/1.73 m^2^. There was no significant difference between the early and late RRT groups in GFR at discharge (98.6 ± 37.2 vs 80.1 ± 42.4 mL/min/1.73 m^2^, respectively, *P *= 0.349).

**Table 2:** Short-term outcome among the early and delayed RRT groups, before and after inverse probability weighting of treatment

	Before weighting			After IPTW weighting			
	
	ED group (*n *= 64)	LD group (*n *= 86)	*P*-value	ED group	LD group	Average treatment effect (95% CI)	*P*-value
Duration of mechanical ventilation, days	12.5 ± 9.8	18.0 ± 13.7	0.049	12.8 ± 7.8	18.9 ± 9.4	-6.02 (-11.50 to -0.54)	0.031
Length of ICU stay, days	18.7 ± 12.5	24.7 ± 17.3	0.093	19.4 ± 11.5	25.4 ± 10.9	-5.97 (-12.91 to 0.976)	0.092
Time on RRT, days	6.9 ± 4.6	8.9 ± 4.6	0.178	6.7 ± 4.9	9.7 ± 6.1	-2.95 (-5.77 to -0.125)	0.041
In-hospital mortality, n (%)	33 (51.5)	67 (77.9)	0.001	51.2	81.7	-30.5 (-14.4 to -45.2)	0.002

Results are presented as mean (± SD) unless stated otherwise. IPTW, inverse probability of treatment weighting; RRT, renal replacement therapy; ED, early dialysis; LD, late dialysis.

### Propensity score analysis

The propensity score model included the variables shown in Table 3. Variables associated with early RRT at ICU admission were lower systolic blood pressure and heart rate. On the day of RRT start, higher sCr and potassium were associated with early RRT initiation. The propensity score model that resulted in the best balance between groups included the variables shown in Table 3.

**Table 3:** Variables included in the propensity score model along with their estimates and standard errors

Variable	Coefficient estimate	Standard error	*P*-value
Lowest sCr pre-RRT, mg/d)	1.803	1.341	0.179
Systolic blood pressure at ICU admission, mmHg	-0.053	0.021	0.010
Heart rate at ICU admission, bpm	-0.054	0.020	0.006
Bicarbonate at ICU admission, mEq/L	-0.123	0.073	0.092
Potassium at ICU admission, mEq/L	0.827	0.407	0.042
GCS	0.221	0.145	0.127
SCr pre-RRT, mg/dL	0.442	0.188	0.019
Hyperkalemia pre-RRT	2.358	1.240	0.057
Azotemia	1.629	1.151	0.157
Oligoanuria	1.464	0.966	0.130

sCr, serum creatinine; RRT, renal replacement therapy; GCS, Glascow Coma Scale.

The propensity score model had good discrimination to predict early RRT, c-index = 0.856, good validation (Hosmer-Lemeshow statistics, *P *= 0.429), and explained 45% of the variability in the data set (*R*^2 ^= 0.45). All demographic, clinical and laboratory variables pre-RRT were well balanced between groups after IPTW, as shown in Table 1. Thus, groups were rendered comparable with regard to all these covariates, and no further adjustment was required for the outcome analysis. In-hospital mortality, ICU LOS and duration of mechanical ventilation were the outcome variables that were comparable between the groups after IPTW weighting. There was a significant 37.3% relative decrease in in-hospital mortality in the early RRT group compared with the late RRT group (Table 2). Moreover, a significant reduction was observed in the duration of mechanical ventilation on a 6-day average, fewer days on RRT-dependence and a trend toward a decrease in ICU LOS in the early RRT group.

## Discussion

In this study, we evaluated the impact of early RRT on mortality in critically ill AKI patients. While previous studies generally used serum urea [8-13] or time from ICU admission to RRT initiation to characterize early RRT [17,18], some recent studies have attempted to define the timing of RRT initiation using RIFLE/AKIN criteria [19-21]. These recent studies have defined early RRT started during the initial stages of RIFLE/AKIN classification. The present study has significant differences from other studies in evaluating early RRT. First, we defined early RRT by time (RRT initiating earlier or later than 24 hours after AKI was diagnosed at its worst stage). Secondly, we studied a highly homogeneous population, excluding patients with AKI-CKD, and performed analysis only considering patients who had achieved AKI stage 3 before initiating RRT. Lastly, AKIN criteria were evaluated by either sCr and/or UO criteria. Using this approach, there was a significant (37.3%) relative decrease in the mortality rate in patients subjected to early RRT.

Several studies have used sUr to define early RRT, with controversial results. In the present study, overall, pre-RRT sUr was similar to levels among patients in early RRT in other studies, suggesting all patients were started relatively early on RRT. Shiao *et al. *[20] demonstrated better survival in patients undergoing abdominal surgery who were submitted to early RRT, defined by the RIFLE criteria. In the aforementioned study, there was a trend towards higher blood urea nitrogen (BUN) in the late RRT group. In the present study, there was no difference in mean sUr levels between patients with early or late RRT (Table 2). This finding demonstrates that early RRT can be associated with better mortality, regardless of pre-RRT urea levels.

To the best of our knowledge, only three studies have evaluated early RRT using the AKIN/RIFLE approach [19-21]. While the previously cited study of patients undergoing abdominal surgery demonstrated a difference in survival rates, the two others, which predominantly included septic patients, were unable to shown any survival benefits of early RRT. When early RRT is defined only as RRT started at earlier AKI stages (that is, RIFLE classification -0 or R), these studies can present some interpretation bias. First, patients with no AKI might be included. As AKI is associated with poor prognosis in critically ill patients, the inclusion of these patients can influence the results. Secondly, patients can achieve a more severe AKI stage by the UO criterion and erroneously remain in the early RRT group. Finally, patients with previous CKD may need RRT earlier during the evolution of an AKI episode due to serious metabolic disorders, mainly when only sCr is available. This can explain the higher prevalence of CKD patients in the groups initiating RRT at RIFLE stages -0 or R [20,21]. In our data, fewer patients starting RRT were classified as AKI stage 3 by sCr. If the UO criterion was not considered, these patients would be classified at earlier AKI stages.

In our study, we attempted to eliminate these pitfalls by separately analyzing only patients in whom CKD could be excluded (patients with baseline sCR < 1.5 mg/dL). Moreover, only patients achieving AKI stage 3 were included, thus selecting a population with the same degree of AKI severity. Finally, we applied both sCr and UO criteria. Recorded urine output disclosed that only 25/240 patients were submitted to RRT before AKI stage 3 was reached. In the study performed by Maccariello *et al. *[19], 52% of patients initiated RRT at RIFLE stages -R or I. In both studies by the National Taiwan University Surgical ICU Associated Renal Failure (NSARF) study group [20,21], approximately half of the patients initiated RRT at RIFLE stages -0 or R. It is probable that these patients would have had more severe AKI stages if the UO criterion had been applied.

We have defined CKD as an inability to achieve sCr ≤ 1.4 mg/dL before RRT. This cutoff was chosen because it is the highest reference level where AKI stage 3 can be achieved only by a three-fold increase in sCr criteria and not by the acute increase of 0.5 mg/dL. This strategy insured that time from renal insult to worst AKI stage is similar in patients with comparable injury severity, according to creatinine kinetics as suggested by Waikar and Bonventre [26].

Timing to RRT was measured after patients achieved AKIN stage 3. We have chosen this method instead of AKI diagnosis at earlier stages because it seems a more practical approach. Not all patients with AKI will need RRT or even progress to AKI stage 3, but it is more likely patients achieving AKIN stage 3 will need RRT. As stated by Lameire *et al. *[27], 'interpretation of studies about early or late dialysis is complicated because there is a loss of the semantic significance of a time-related event' and that 'conclusions on the impact of early versus late initiation of dialysis would be only justified when, at the moment patients fulfilled the criteria for dialysis, one group started RRT immediately while for the other group dialysis was delayed.' In the present methodology, all patients achieved AKI at stage 3 and RRT was a time-related event. We suggest that one parameter used to initiate RRT in critically ill patients can be when stage 3 is reached and renal function recuperation within a short time has been ruled out. This does not eliminate the possibility of starting RRT at early stages when associated with severe metabolic disorders and/or refractory hypervolemia.

Although showing promising results in terms of performing early RRT in critically ill patients, this study evaluated only AKIN stage 3, so the influence of RRT on AKI earlier stages was not evaluated. This limitation is counterbalanced by considering UO. When using the UO criterion, many patients classified AKIN 3 would be otherwise classified AKIN 1 or 2, using only sCr alone. Including UO criteria could explain why a minority of patients needed RRT before achieving AKIN stage 3. A second limitation is that excluding patients who could not achieve a sCr lower than 1.4 mg/dL, might mean that patients with community-acquired AKI have been considered as having CKD. In these patients, it would not be possible to precisely determine when AKI reached stage 3, making it difficult to classify them into early or late RRT groups. Another limitation is the lack of data on fluid balance, making it especially difficult to explore reasons for reduced mechanical ventilation time in early RRT patients. Although it is a retrospective study, we have tried to balance all variables that could influence the decision to initiate RRT earlier, using the propensity score and the IPTW method to balance variables. Finally, the relatively small number of patients is counterbalanced by the homogeneity of this population, including only patients with pure AKI.

## Conclusions

Using a time-based approach from AKIN stage 3 by UO or sCr to RRT initiation, this study demonstrated a reduced mortality rate and reduced need for mechanical ventilation among critically ill AKI patients receiving RRT in the first 24 h after diagnosis of AKI stage 3, by applying both sCr and UO criteria. Patients with AKI-CKD can influence results of studies that use AKIN/RIFLE classification to define early RRT. We suggest that in patients with stage 3 AKI, RRT must be considered in less than 24 h.

## Key messages

• Urinary output is an important criterion for staging AKI and can be useful in determining the timing of RRT.

• Patients with previous CKD can influence the results of studies when classifying early RRT by AKI stage.

• Performing RRT in the first 24 h of AKIN stage 3 diagnosis reduced in-hospital mortality and mechanical ventilation time.

## Abbreviations

AKI-CKD: acute-on-chronic kidney disease; AKIN: Acute Kidney Injury Network; APACHE II: Acute Physiology and Chronic Health Evaluation II; CKD: chronic kidney disease; CKD-EPI: chronic kidney disease epidemiology collaboration; ED: early dialysis; GCS: Glasgow Coma Scale; GFR: glomerular filtration rate; IPTW: inverse probability of treatment weighting; LD: late dialysis; LOS: length of stay; NSRAF: National Taiwan University Surgical ICU Associated Renal Failure; RIFLE: risk, injury, failure, loss of kidney function, end stage renal failure; RRT: renal replacement therapy; SAPS III: Simplified Acute physiology Score III; sCr: serum creatinine; SLED: sustained low efficiency dialysis; sUr: serum urea; UO: urinary output.

## Competing interests

All the authors declare no competing interests.

## Authors' contributions

ABL conceived the study, performed statistical analysis and participated in manuscript writing. EM participated in data analysis and manuscript revision. TTL participated in data collection and manuscript writing and revision. SMP conceived the study and participated in data collection and manuscript review. SRCB, PHSP, ASG, FRM, IMMS and LMA participated in data collection and manuscript writing. All authors read and approved the final manuscript.

## References

[B1] Uchino S Kellum JA Bellomo R Doig GS Morimatsu H Morgera S Schetz M Tan I Bouman C Macedo E Gibney N Tolwani A Ronco C Beginning and Ending Supportive Therapy for the Kidney (BEST Kidney) Investigators Acute renal failure in critically ill patients: a multinational, multicenter study JAMA 2005 17 813 818 10.1001/jama.294.7.813 16106006

[B2] Hoste EAJ Schurgers M Epidemiology of acute kidney injury: how big is the problem? Crit Care Med 2008 17 S146 S151 10.1097/CCM.0b013e318168c590 18382186

[B3] Hoste EAJ Clermont G Kersten A Venkataraman R Angus DC De Bacquer D Kellum JA RIFLE criteria for acute kidney injury are associated with hospital mortality in critically ill patients: a cohort analysis Crit Care 2006 17 R73 10.1186/cc4915 16696865 PMC1550961

[B4] Murugan R Kellum JA Acute kidney injury: what's the prognosis? Nat Rev Nephrol 2011 17 209 217 10.1038/nrneph.2011.13 21343898 PMC3547642

[B5] Karvellas CJ Farhat MR Sajjad I Mogensen SS Leung AA Wald R Bagshaw SM A comparison of early versus late initiation of renal replacement therapy in critically ill patients with acute kidney injury: a systematic review and meta-analysis Crit Care 2011 17 R72 10.1186/cc10061 21352532 PMC3222005

[B6] Seabra VF Balk EM Liangos O Sosa MA Cendoroglo M Jaber BL Timing of renal replacement therapy initiation in acute renal failure: a meta-analysis Am J Kidney Dis 2008 17 272 284 10.1053/j.ajkd.2008.02.371 18562058

[B7] Liu KD Himmelfarb J Paganini E Ikizler TA Soroko SH Mehta RL Chertow GM Timing of initiation of dialysis in critically ill patients with acute kidney injury Clin J Am Soc Nephrol 2006 17 915 919 10.2215/CJN.01430406 17699307

[B8] Bagshaw SM Uchino S Bellomo R Morimatsu H Morgera S Schetz M Tan I Bouman C Macedo E Gibney N Tolwani A Oudemans-van Straaten HM Ronco C Kellum JA Beginning and Ending Supportive Therapy for the Kidney (BEST Kidney) Investigators Timing of renal replacement therapy and clinical outcomes in critically ill patients with severe acute kidney injury J Crit Care 2009 17 129 140 10.1016/j.jcrc.2007.12.017 19272549

[B9] Gettings LG Reynolds HN Scalea T Outcome in post-traumatic acute renal failure when continuous renal replacement therapy is applied early vs. late Intensive Care Med 1999 17 805 813 10.1007/s001340050956 10447537

[B10] Demirkiliç U Kuralay E Yenicesu M Cağlar K Oz BS Cingöz F Günay C Yildirim V Ceylan S Arslan M Vural A Tatar H Timing of replacement therapy for acute renal failure after cardiac surgery J Card Surg 2004 17 17 20 10.1111/j.0886-0440.2004.04004.x 15108784

[B11] Wu V-C Ko W-J Chang H-W Chen Y-S Chen Y-W Chen Y-M Hu F-C Lin Y-H Tsai P-R Wu K-D Early renal replacement therapy in patients with postoperative acute liver failure associated with acute renal failure: effect on postoperative outcomes J Am Coll Surg 2007 17 266 276 10.1016/j.jamcollsurg.2007.04.006 17660073

[B12] Manché A Casha A Rychter J Farrugia E Debono M Early dialysis in acute kidney injury after cardiac surgery Interact Cardiovasc Thorac Surg 2008 17 829 832 10.1510/icvts.2008.181909 18603545

[B13] Carl DE Grossman C Behnke M Sessler CN Gehr TWB Effect of timing of dialysis on mortality in critically ill, septic patients with acute renal failure Hemodial Int 2010 17 11 17 10.1111/j.1542-4758.2009.00407.x 20377649

[B14] Bouman CSC Oudemans-Van Straaten HM Tijssen JGP Zandstra DF Kesecioglu J Effects of early high-volume continuous venovenous hemofiltration on survival and recovery of renal function in intensive care patients with acute renal failure: a prospective, randomized trial Crit Care Med 2002 17 2205 2211 10.1097/00003246-200210000-00005 12394945

[B15] Sugahara S Suzuki H Early start on continuous hemodialysis therapy improves survival rate in patients with acute renal failure following coronary bypass surgery Hemodial Int 2004 17 320 325 10.1111/j.1492-7535.2004.80404.x 19379436

[B16] Elahi MM Lim MY Joseph RN Dhannapuneni RRV Spyt TJ Early hemofiltration improves survival in post-cardiotomy patients with acute renal failure Eur J Cardiothorac Surg 2004 17 1027 1031 10.1016/j.ejcts.2004.07.039 15519198

[B17] Andrade L Cleto S Seguro AC Door-to-dialysis time and daily hemodialysis in patients with leptospirosis: impact on mortality Clin J Am Soc Nephrol 2007 17 739 744 10.2215/CJN.00680207 17699490

[B18] Iyem H Tavli M Akcicek F Büket S Importance of early dialysis for acute renal failure after an open-heart surgery Hemodial Int 2009 17 55 61 10.1111/j.1542-4758.2009.00347.x 19210279

[B19] Maccariello E Soares M Valente C Nogueira L Valença RVR Machado JES Rocha E RIFLE classification in patients with acute kidney injury in need of renal replacement therapy Intensive Care Med 2007 17 597 605 10.1007/s00134-007-0535-0 17310365

[B20] Shiao C-C Wu V-C Li W-Y Lin Y-F Hu F-C Young G-H Kuo C-C Kao T-W Huang D-M Chen Y-M Tsai P-R Lin S-L Chou N-K Lin T-H Yeh Y-C Wang C-H Chou A Ko W-J Wu K-D National Taiwan University Surgical Intensive Care Unit-Associated Renal Failure Study Group Late initiation of renal replacement therapy is associated with worse outcomes in acute kidney injury after major abdominal surgery Crit Care 2009 17 R171 10.1186/cc8147 19878554 PMC2784403

[B21] Chou YH Huang TM Wu VC Wang CY Shiao CC Lai CF Tsai HB Chao CT Young GH Wang WJ Kao TW Lin SL Han YY Chou A Lin TH Yang YW Chen YM Tsai PR Lin YF Huang JW Chiang WC Chou NK Ko WJ Wu KD Tsai TJ NSARF Study Group Impact of timing of renal replacement therapy initiation on outcome of septic acute kidney injury Crit Care 2011 17 R134 10.1186/cc10252 21645350 PMC3219003

[B22] Bone RC Balk RA Cerra FB Dellinger RP Fein AM Knaus WA Schein RM Sibbald WJ ACCP/SCCM Consensus Conference Committee Definitions for sepsis and organ failure and guidelines for the use of innovative therapies in sepsis. The ACCP/SCCM Consensus Conference Committee. American College of Chest Physicians/Society of Critical Care Medicine. 1992 Chest 2009 17 e28 10.1378/chest.09-2267 1303622

[B23] Austin PC The performance of different propensity-score methods for estimating relative risks J Clin Epidemiol 2008 17 537 545 10.1016/j.jclinepi.2007.07.011 18471657

[B24] Harder VS Stuart EA Anthony JC Propensity score techniques and the assessment of measured covariate balance to test causal associations in psychological research Psychol Methods 2010 17 234 249 20822250 10.1037/a0019623 PMC2936698

[B25] The R project for statistical computing http://www.r-project.org

[B26] Waikar SS Bonventre JV Creatinine kinetics and the definition of acute kidney injury J Am Soc Nephrol 2009 17 672 679 10.1681/ASN.2008070669 19244578 PMC2653692

[B27] Lameire N Vanbiesen W Vanholder R When to start dialysis in patients with acute kidney injury? When semantics and logic become entangled with expectations and beliefs Crit Care 2011 17 171 10.1186/cc10280 21861864 PMC3387587

